# Evaluation of the impact of once weekly dulaglutide on patient-reported outcomes in Japanese patients with type 2 diabetes: comparisons with liraglutide, insulin glargine, and placebo in two randomized studies

**DOI:** 10.1186/s12955-017-0696-7

**Published:** 2017-06-12

**Authors:** Shuichi Suzuki, Tomonori Oura, Masakazu Takeuchi, Kristina S. Boye

**Affiliations:** 1 Medicines Development Unit Japan, Eli Lilly Japan K.K, Sannomiya Plaza Bldg. 7-1-5, Isogamidori, Chuo-ku, Kobe, 651-0086 Japan; 2 Global Patient Outcomes and Real World Evidence, Eli Lilly and Company, Lilly Corporate Center, Indianapolis, IN 46285 USA

**Keywords:** Type 2 diabetes, Glucagon-like peptide-1, Patient-reported outcomes, Dulaglutide

## Abstract

**Background:**

Standardized patient-reported outcome (PRO) questionnaires can be utilized to evaluate treatment satisfaction (subjective evaluation of treatment) in patients with type 2 diabetes (T2D). These outcomes are important because they may affect patient adherence and overall study results.

**Methods:**

PROs were evaluated in two randomized 26-week clinical trials in Japanese patients with T2D taking dulaglutide 0.75 mg (dulaglutide) once weekly; comparators were once-daily liraglutide (0.9 mg/day) and once-weekly placebo in one study and once-daily insulin glargine (glargine) in the other study. The Perceptions About Medications-Diabetes 21 Questionnaire - Japanese version (PAM-D21-J) and the Injectable Diabetes Medication Questionnaire - Japanese version (IDMQ-J) were completed by patients in both studies. These measures were both considered exploratory endpoints. All scale scores range from 0 to 100, with higher scores reflecting better outcomes.

**Results:**

Patients reported that dulaglutide was more convenient and flexible than liraglutide (PAM-D21-J Convenience/Flexibility subscale: dulaglutide least-square mean [LSM], 84.58; liraglutide LSM, 78.94; *p* = .026), and that they were more satisfied with dulaglutide than with liraglutide (IDMQ-J Satisfaction subscale: dulaglutide, 75.24; liraglutide, 69.53; *p* = .012). Patients also reported that dulaglutide was more convenient and flexible than glargine (PAM-D21-J Convenience/Flexibility subscale: dulaglutide, 87.89; glargine, 79.22; *p* < .001), and that they were more satisfied with dulaglutide than with glargine (IDMQ-J Satisfaction subscale: dulaglutide, 78.86; glargine, 69.66; *p* < .001), and felt dulaglutide was more effective than glargine, with fewer symptoms and adverse events (PAM-D21-J Perceived Effectiveness subscale: dulaglutide, 77.61; glargine, 67.22; *p* < .001; Emotional Effects subscale: dulaglutide, 93.02; glargine, 89.55; *p* = .017; IDMQ-J Blood Glucose Control subscale: dulaglutide, 76.33; glargine, 67.57; *p* < .001). In addition, patients responded that dulaglutide was superior to placebo in the PAM-D21-J Convenience/Flexibility, Perceived Effectiveness, and Emotional Effects subscales and all IDMQ-J subscales (Satisfaction, Ease of Use, Lifestyle Impact, Blood Glucose Control).

**Conclusions:**

Overall, after 26 weeks of once-weekly dulaglutide administration in Japanese patients with T2D, PROs were generally positive versus the three comparator treatments (liraglutide, glargine, and placebo), suggesting increased treatment satisfaction through better blood glucose control and convenience/flexibility and reduced negative emotional effects of diabetes.

**Trial registration:**

ClinicalTrials.gov (monotherapy study: NCT01558271, registered March 12, 2012; combination therapy study: NCT01584232, registered April 23, 2012).

**Electronic supplementary material:**

The online version of this article (doi:10.1186/s12955-017-0696-7) contains supplementary material, which is available to authorized users.

## Background

Diabetes is a chronic metabolic disorder and is a major public health threat, with more than 415 million people globally and 7.2 million people in Japan diagnosed with the disease [1]. In international and Japanese guidelines, treatment of type 2 diabetes (T2D) includes diet, physical exercise, and weight control followed by oral and/or injectable therapies [2, 3].

Due to the progressive nature of the disease, patients require the right treatment at the right time based on their clinical and psychological conditions; to achieve diabetes treatment goals in clinical practice, patients must be fully engaged with their therapy [2, 3]. When choosing a treatment strategy, clinicians must strive for good clinical and physical outcomes for patients while also considering patients’ psychosocial well-being [4]. For example, preliminary evidence suggests that various clinical and psychosocial factors can become barriers to injection therapy [5, 6]. Based on responses of both patients and physicians in a study in Japan and other key research, barriers to the initiation of insulin treatment include fear, pain, and inconvenience associated with injections; concerns about weight gain and cost; and fear that the disease will continue to worsen [5, 7, 8].

In clinical trials, patient-reported outcome (PRO) measures complement clinical measures by providing information beyond traditional efficacy and safety parameters [9]. PRO questionnaires can be used to examine whether drug differences other than in clinical efficacy have an impact on outcomes that may be important to patients. For example, patient satisfaction, the subjective evaluation of treatment (including outcomes and processes), can be assessed with PRO questionnaires. Satisfaction is an important outcome in diabetes treatment because it may affect treatment compliance and adherence [10].

**Fig. 1:**
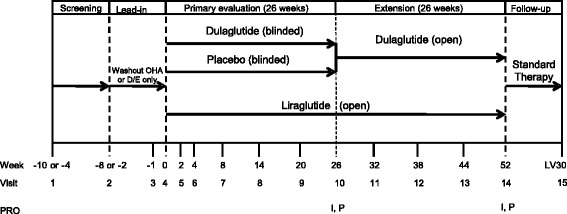
Study Design for the Monotherapy Study. *D/E* diet and exercise; *I* Injectable Diabetes Medication Questionnaire - Japanese version; *LV30* follow-up visit 30 days after last study visit; *OHA* oral hypoglycemic agent; *P* Perceptions About Medications-Diabetes 21 Questionnaire - Japanese version; *PRO* Patient-Reported Outcomes

**Fig. 2:**
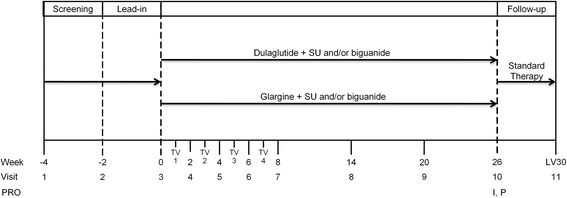
Study Design for the Combination Therapy Study. *I* Injectable Diabetes Medication Questionnaire - Japanese version; *LV30* follow-up visit 30 days after last study visit; *P* Perceptions About Medications-Diabetes 21 Questionnaire - Japanese version; *PRO* patient-reported outcomes; *SU* sulfonylurea; *TV* telephone visit

Dulaglutide is a long-acting human glucagon-like peptide-1 (GLP-1) receptor agonist that is administered once weekly via subcutaneous injection. It has been approved for the treatment of T2D in the United States and European Union at doses of 0.75 and 1.5 mg and in Japan at a dose of 0.75 mg [11–13]. PROs were used in two randomized, phase 3 clinical trials of patients treated with once-weekly dulaglutide 0.75 mg (dulaglutide) in Japan to assess treatment satisfaction and psychological perception. One trial (henceforth referred to as “the monotherapy study”) was a 52-week (primary endpoint at 26 weeks) study in which patients received double-blind dulaglutide monotherapy or placebo or open-label once-daily liraglutide 0.9 mg monotherapy; after 26 weeks, patients receiving placebo were switched to dulaglutide (ClinicalTrials.gov: NCT01558271) (Fig. 1) [14]. The other trial (hereafter referred to as “the combination therapy study”) was a 26-week open-label study in which dulaglutide was compared to once-daily insulin glargine (glargine) in patients also treated with sulfonylureas and/or biguanides (ClinicalTrials.gov: NCT01584232) (Fig. 2) [15].

This is the first known published report of treatment satisfaction in Asian patients with T2D treated with dulaglutide as of April 2017, although PROs were assessed in some Asian patients in the global AWARD-3 and AWARD-5 studies [16]. The two studies reported here are the only phase 3 studies of dulaglutide in Japan that included PRO measures; the impacts of once-weekly injectable treatment on patients’ satisfaction and perceptions of treatment were of key interest.

## Methods

### Study design

Dulaglutide was administered in both studies using a prefilled syringe (a device specific to clinical trials) because the device later marketed was not available at the time the clinical studies were conducted.

In the monotherapy study, eligible patients were randomized to treatment in a 4:2:1 ratio (dulaglutide:liraglutide:placebo) [14]. Randomization was stratified by prestudy oral hypoglycemia agent (OHA) status (yes/no), body mass index (BMI; <25 and ≥25 kg/m^2^), and glycated hemoglobin (HbA1c; ≤8.5 or >8.5%). Patients and investigators were masked to dulaglutide and placebo treatment assignments but were not masked to liraglutide treatment assignment. Liraglutide was administered using a pen injector (marketed product). Placebo was administered using the same prefilled syringe used for dulaglutide administration.

In the combination therapy study, eligible patients were randomized to treatment in a 1:1 ratio (dulaglutide:glargine) [15]. Randomization was stratified by concomitant OHA regimen (sulfonylureas only, biguanides only, or sulfonylurea and biguanide), BMI (<25 and ≥25 kg/m^2^), and HbA1c (≤8.5 and >8.5%). An open-label design was used, and participants, investigators, and site staff were not masked to treatment assignments. Glargine was administered using a prefilled disposable pen (marketed product).

The primary endpoint of both studies was change from baseline in HbA1c (%) at week 26 [14, 15].

### Patients

In the monotherapy study, eligible patients were Japanese males or females with T2D aged 20 years or older with BMIs ≥18.5 and ≤35.0 kg/m^2^ and HbA1c ≥7.0 and ≤10.0% confirmed at randomization who were OHA-naïve (diet and exercise only) or had discontinued OHA monotherapy [14].

In the combination therapy study, eligible patients were Japanese males or females with T2D aged 20 years or older with a BMI ≥18.5 and <35.0 kg/m^2^ and HbA1c at screening ≥7.0 and ≤10.0% who were taking stable doses of sulfonylureas and/or biguanides [15].

### Patient-reported outcome instruments

PRO measures were considered exploratory endpoints in the studies.

The Perceptions About Medications Diabetes 21 Questionnaire - Japanese version (PAM-D21-J) and the Injectable Diabetes Medication Questionnaire - Japanese version (IDMQ-J) were completed by patients at week 26 in both studies (English-translated versions provided in Additional files 1 and 2 for PAM-D21-J and IDMQ-J, respectively). The PAM-D21-J was developed to assess perceptions about diabetes medications, focusing on frequency, amount, timing, and effectiveness of the drugs as well as on the physical and emotional side effects. It was based on the Perception About Medications-Diabetes Questionnaire (PAM-D) [17]. This scale comprises 21 questions forming four subscales: Convenience/Flexibility (three items), Perceived Effectiveness (three items), Emotional Effects (five items), and Physical Effects (10 items). Item scores for Convenience/Flexibility, Perceived Effectiveness, and Physical Effects were reversed prior to statistical analysis so that for all four subscales a higher score represented a more favorable state. Subscale scores were derived by summing the item scores. The scores for all four subscales were linearly transformed to a 0 to 100 scale using the following formula:

$$ \mathrm{subscale}\ 100\ \mathrm{score}=\frac{\mathrm{observed}\ \mathrm{score}\kern0.5em \hbox{-} \kern0.5em \mathrm{minimum}\ \mathrm{possible}\ \mathrm{value}}{\mathrm{maximum}\ \mathrm{possible}\ \mathrm{value}\kern0.5em \hbox{-} \kern0.5em \mathrm{minimum}\ \mathrm{possible}\ \mathrm{value}}\times 100 $$

The IDMQ-J was developed to assess treatment satisfaction, ease of use, lifestyle impact, and blood glucose control by injectable diabetes medication, such as GLP-1 receptor agonists, based on the Insulin Delivery System Questionnaire, Japanese version (IDSQ-J) [18]. This scale comprises 11 questions. The scores for items 1 and 2a to 2f range from 1 to 7 and for items 3a to 3c range from 1 to 6, and the score for item 4 ranges from 1 to 5. Satisfaction score (item 1), Ease of Use score (items 2a and 2e), Lifestyle Impact score (items 2c, 2d, and 2f), and Blood Glucose Control score (items 2b, 3a [reverse], and 3b) were derived by summing the corresponding scores and were linearly transformed to a 0 to 100 scale using the formula described previously for the PAM-D21-J. A higher score represented a more favorable state for all four subscales. Because the PAM-D21-J and IDMQ-J have been developed recently, neither instrument has been fully psychometically validated or published elsewhere at this time.

In addition, to assess baseline characteristics of study participants in terms of general health status, the five-response-level EQ-5D-5L questionnaire was administered at baseline in the monotherapy study. The EQ-5D-5L is a widely used, validated generic questionnaire that assesses health-related quality of life [19]. It consists of two parts. The first part assesses five dimensions associated with quality of life (mobility, self-care, usual activities, pain/discomfort, and anxiety/depression), which are combined to produce a single index/utility score based on the up-to-date value set of the Japanese population from Ikeda et al. [20]; for this analysis this scoring was done post hoc. For the index score, 1 is the best score (higher scores indicate greater health), a score of 0 represents death, and scores less than 0 represent conditions perceived as worse than death. The second part of the questionnaire consists of a 100-mm visual analog scale (VAS) on which the patient rates his or her perceived health state on that day from 0 mm (worst imaginable health state) to 100 mm (best imaginable health state).

### Statistical analysis

Statistical analyses were conducted on the Full Analysis Set (FAS) populations in both studies, defined as all randomized patients who received at least one dose of study medication, with last observation carried forward (LOCF) used to impute missing postbaseline values.

In both studies, the responses to the items of the PAM-D21-J were summarized individually by treatment as counts and percentages, and the PAM-D21-J subscale scores at 26 weeks were described and compared between treatment groups using an analysis of variance (ANOVA) model. The ANOVA included treatment, prestudy therapy, and BMI group at baseline as fixed effects. In both studies, the responses to the items of the IDMQ-J were summarized individually by treatment as counts and percentages. The IDMQ-J subscale scores at 26 weeks were described and compared between treatment groups in each study using the same ANOVA model described above for the PAM-D21-J. Least-square means (LSMs) for treatment differences and *p*-values for pairwise comparisons between treatments based on the ANOVA model were reported for both instruments. All analyses were prespecified in the study analysis plan.

## Results

### Baseline characteristics

Patient demographics at baseline for both studies are summarized in Table 1. In both studies, the majority of patients (>70%) were male, and the mean age was approximately 57 years. Mean duration of diabetes was approximately 6 to 7 years in the monotherapy study and approximately 9 years in the combination therapy study. Mean HbA1c at baseline was approximately 8% in both studies.

**Table 1:** Patient characteristics at baseline, Full Analysis Set

	Monotherapy Study			Combination Therapy Study	
	Dulaglutide 0.75 mg (*N* = 280)	Liraglutide 0.9 mg (*N* = 137)	Placebo (*N* = 70)	Dulaglutide 0.75 mg (*N* = 181)	Insulin Glargine (*N* = 180)
Females, *n* (%)	52 (19%)	24 (18%)	15 (21%)	56 (31%)	47 (26%)
Age (yrs), mean (SD)	57.2 (9.6)	57.9 (10.4)	57.7 (8.3)	57.5 (10.5)	56.1 (11.3)
Weight (kg), mean (SD)	71.3 (12.5)	70.2 (12.5)	69.3 (11.6)	70.9 (13.7)	71.1 (13.8)
BMI (kg/m^2^), mean (SD)	25.6 (3.6)	25.5 (3.5)	25.2 (3.2)	26.1 (3.6)	25.9 (3.9)
Diabetes duration (yrs), mean (SD)	6.8 (5.6)	6.3 (6.0)	6.3 (5.1)	8.9 (6.7)	8.8 (6.1)
HbA1c (%), mean (SD)	8.2 (0.8)	8.1 (0.9)	8.2 (0.8)	8.1 (0.8)	8.0 (0.9)
Receiving OHA at screening, *n* (%)	94 (34%)	48 (35%)	22 (31%)	181 (100%)	180 (100%)
OHA-naive, *n* (%)	186 (66%)	89 (65%)	48 (69%)	0 (0%)	0 (0%)
Concomitant OHA at baseline, *n* (%)					
Sulfonylureas only	NA	NA	NA	34 (19%)	33 (18%)
Biguanides only	NA	NA	NA	64 (35%)	66 (37%)
Sulfonylureas and biguanides	NA	NA	NA	83 (46%)	81 (45%)
EQ-5D-5L Japan Index, mean (SD)	0.97 (0.06)	0.97 (0.06)	0.96 (0.08)	NA	NA
EQ-5D VAS, mean (SD)	82.6 (12.7)	81.0 (11.4)	79.3 (14.2)	NA	NA

*BMI* Body mass index, *HbA1c* Glycosylated hemoglobin A1c, *NA* Not applicable, *OHA* Oral hypoglycemic agent, *SD* Standard deviation, *VAS* Visual analog scale

EQ-5D-5L data have not been often collected or reported in Japanese patients with T2D. EQ-5D-5L Japan Index and VAS scores at baseline by treatment in the monotherapy study are presented in Table 1. Across all three treatment groups, the mean EQ-5D-5L Japan Index score at baseline was approximately 0.97, and the mean EQ-5D VAS was approximately 82.

### Key results

At week 26 in the monotherapy study, dulaglutide was superior to placebo for HbA1c change from baseline (the primary objective of the study; *p* < .001) [14]. Dulaglutide was also noninferior (margin 0.4%), but not superior, to once-daily liraglutide. The LSM (standard error [SE]) changes in HbA1c from baseline to week 26 were −1.43% (0.05) for dulaglutide, −1.33% (0.07) for liraglutide, and 0.14% (0.10) for placebo. The LSM differences were −1.57% (95% confidence interval [CI] [−1.79% to −1.35%]) between dulaglutide and placebo and −0.10% (95% CI [−0.27 to 0.07%]) between dulaglutide and liraglutide. The incidence of hypoglycemia through 26 weeks was 2.1% for dulaglutide, 1.5% for liraglutide, and 1.4% for placebo.

At week 26 in the combination therapy study, dulaglutide was superior to glargine for HbA1c change from baseline (the primary objective of the study; *p* < .001) [15]. The LSM (SE) changes in HbA1c from baseline to week 26 were −1.44% (0.05) for dulaglutide and −0.90% (0.05) for glargine. The LSM difference between dulaglutide and glargine was −0.54% (95% CI [−0.67% to −0.41%]). The incidence of hypoglycemia through 26 weeks was 26.0% for dulaglutide and 47.8% for glargine.

### PRO results at week 26

**Fig. 3:**
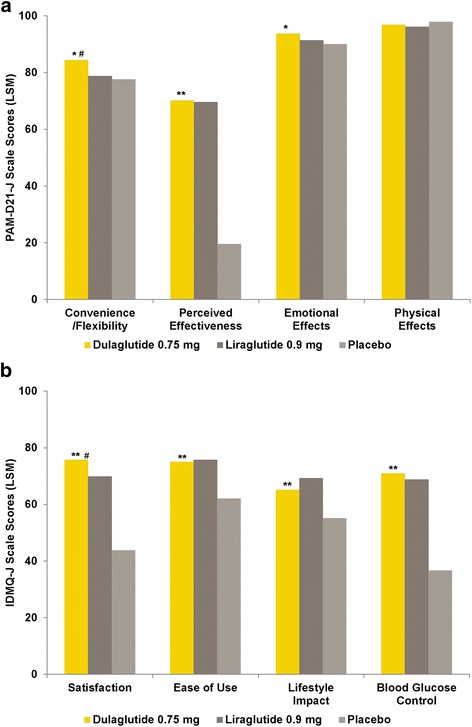
Results of the PAM-D21-J and IDMQ-J at Week 26 in the Monotherapy Study. (**a**) PAM-D21-J at week 26 (LSM scores by treatment). (**b**) IDMQ-J at week 26 (LSM scores by treatment). *IDMQ-J* Injectable Diabetes Medication Questionnaire - Japanese version; *LSM* least-squares mean; *PAM-D21-J* Perceptions About Medications-Diabetes 21 Questionnaire - Japanese version. Dulaglutide and placebo administered once weekly; liraglutide administered once daily. For all subscales higher scores represent a more favorable state. *p < .05 vs. placebo, **p < .001 vs. placebo, ^#^ *p* < .05 vs. liraglutide

**Fig. 4:**
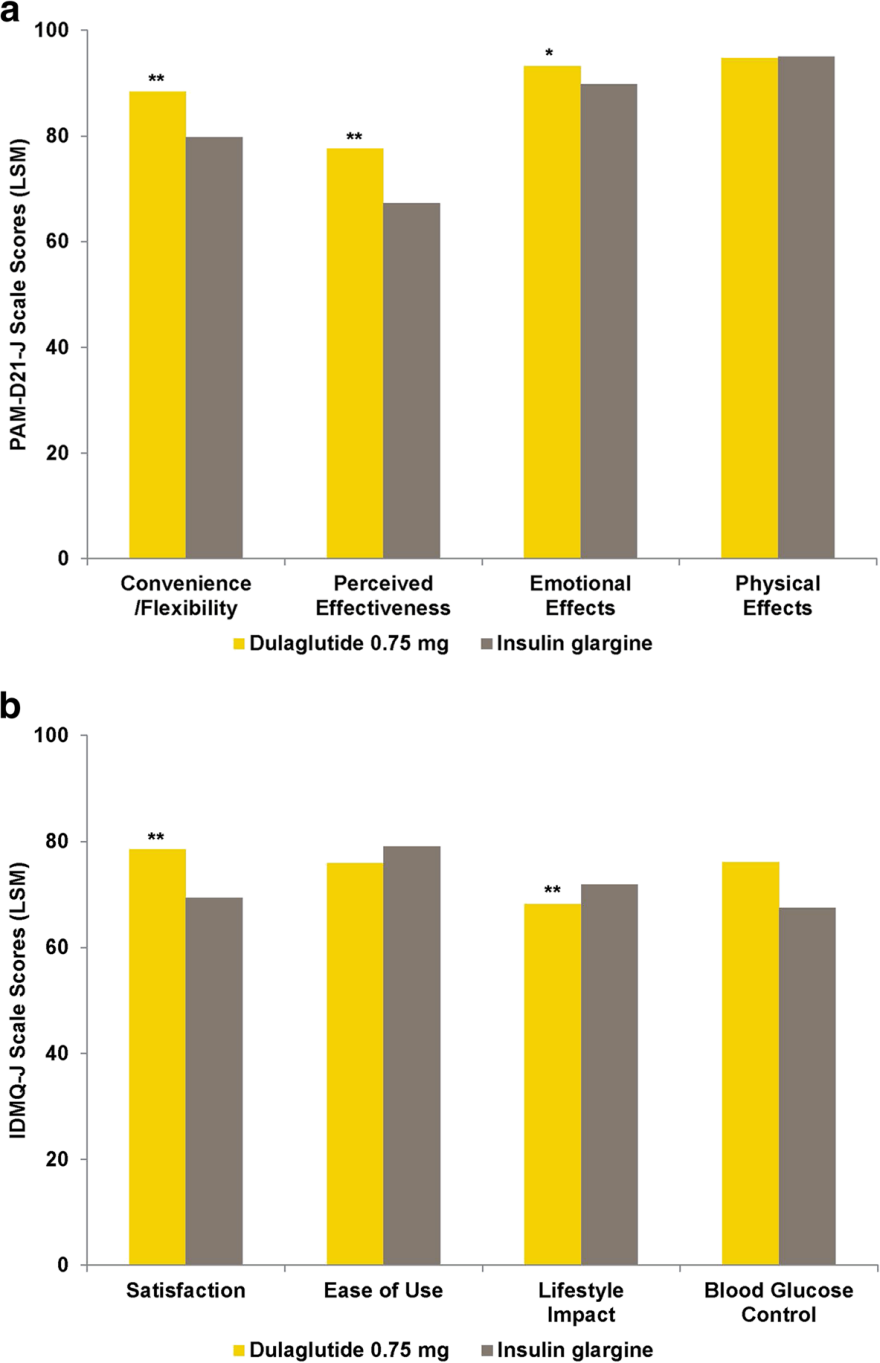
Results of the PAM-D21-J and IDMQ-J at Week 26 in the Combination Therapy Study. (**a**) PAM-D21-J at week 26 (LSM scores by treatment). (**b**) IDMQ-J at week 26 (LSM scores by treatment). *IDMQ-J* Injectable Diabetes Medication Questionnaire - Japanese version; LSM, least-squares mean; *PAM-D21-J* Perceptions About Medications-Diabetes 21 Questionnaire - Japanese version. Dulaglutide administered once weekly; insulin glargine administered once daily. For all subscales higher scores represent a more favorable state. *p < .05 vs. insulin glargine, **p < .001 vs. insulin glargine

LSM endpoint scores of the PAM-D21-J and IDMQ-J subscales at week 26 (LOCF) are presented in Fig. 3 for the monotherapy study and in Fig. 4 for the combination therapy study.

#### Dulaglutide versus liraglutide (monotherapy study)

Dulaglutide was superior to liraglutide in the PAM-D21-J Convenience/Flexibility subscale (dulaglutide vs. liraglutide LSM, 84.58 vs. 78.94; *p* = .026). No significant treatment difference was observed in the Perceived Effectiveness (69.50 vs. 69.07; *p* = .874), Emotional Effects (93.84 vs. 91.48; *p* = .073), or Physical Effects (96.79 vs. 96.13; *p* = .336) subscales.

Dulaglutide was superior to liraglutide in the IDMQ-J Satisfaction subscale (dulaglutide vs. liraglutide LSM, 75.24 vs. 69.53; *p* = .012). No significant treatment difference was observed in the Ease of Use (75.07 vs. 75.80; *p* = .704), Lifestyle Impact (64.94 vs. 69.15; *p* = .053), or Blood Glucose Control (70.55 vs. 68.51; *p* = .300) subscales.

#### Dulaglutide versus placebo (monotherapy study)

Dulaglutide was superior to placebo in the PAM-D21-J Convenience/Flexibility (dulaglutide vs. placebo LSM, 84.58 vs. 77.85; *p* = .040), Perceived Effectiveness (69.50 vs. 18.71; *p* < .001), and Emotional Effects (93.84 vs. 90.13; *p* = .029) subscales and all IDMQ-J subscales (Satisfaction [75.24 vs. 43.19; *p* < .001], Ease of Use [75.07 vs. 62.13; *p* < .001], Lifestyle Impact [64.94 vs. 54.82; *p* < .001], Blood Glucose Control [70.55 vs. 36.12; *p* < .001]). No significant treatment difference was observed in the PAM-D21-J Physical Effects subscale (96.79 vs. 97.74; *p* = .290).

#### Dulaglutide versus glargine (combination therapy study)

Dulaglutide was superior to glargine in the PAM-D21-J Convenience/Flexibility (dulaglutide LSM, 87.89; glargine LSM, 79.22; *p < .*001), Perceived Effectiveness (77.61 vs. 67.22; *p < .*001), and Emotional Effects (93.02 vs. 89.55; *p = .*017) subscales. No significant treatment difference was observed in the Physical Effects subscale (94.79 vs. 95.02; *p = .*798).

Dulaglutide was superior to glargine in the IDMQ-J Satisfaction (dulaglutide vs. glargine LSM, 78.86 vs. 69.66; *p* < .001) and Blood Glucose Control (76.33 vs. 67.57; *p* < .001) subscales. No significant treatment difference was observed in the Ease of Use (75.77 vs. 78.93; *p* = .090) or Lifestyle Impact (68.00 vs. 71.58; *p* = .108) subscales.

## Discussion

The two instruments presented here (PAM-D21-J and IDMQ-J) were used as exploratory measures in the studies to evaluate once-weekly injectable treatment with dulaglutide in Japanese patients with T2D.

Overall in two randomized studies of once-weekly dulaglutide in Japan, 26 weeks of treatment with dulaglutide resulted in greater treatment satisfaction and better perception of the treatment compared to liraglutide, glargine, and placebo. The questionnaires evaluated in these studies were chosen based on key attributes of the medications administered in the studies (eg, clinical characteristics of the active ingredients, administration frequency, and injection device).

### Dulaglutide versus liraglutide (monotherapy study)

Dulaglutide was superior to liraglutide in the PAM-D21-J Convenvience/Flexibility subscale. Dulaglutide was also superior to liraglutide in the IDMQ-J Satisfaction subscale; the reduced frequency of administration for dulaglutide (once weekly) vs. liraglutide (once daily) might be one of the reasons for this finding [21, 22]. However, there was no significant difference in the IDMQ-J Ease of Use subscale. The PAM-D21-J Convenience/Flexibility subscale and the IDMQ-J Ease of Use subscale assess similar concepts, but it seemed that patients answered differently depending on the exact wording of the questions: the PAM-D21-J Convenience/Flexibility subscale includes a specific question about the frequency of administration, whereas the IDMQ-J Ease of Use subscale includes an abstract question about convenience but does not include any questions about the frequency of administration.

### Dulaglutide versus placebo (monotherapy study)

Dulaglutide was superior to placebo in all PAM-D21-J subscales except for the Physical Effects subscale, indicating that patients were more satisfied overall with dulaglutide treatment compared to placebo. The Physical Effects subscale scores in both groups were high, indicating that patients in both groups did not experience negative physical consequences commonly observed with existing diabetes treatments, such as weight gain, abdominal bloating, and nausea.

Despite the fact that dulaglutide and placebo were both administered once weekly with the same device in a double-blind fashion, dulaglutide significantly improved the PAM-D21-J Convenience/Flexibility and IDMQ-J Ease of Use subscales compared to placebo. Although there were no items in these subscales directly related to glycemic control, patients’ satisfaction with their glycemic control during the study may have subconsciously affected their answers to questions about the convenience, flexibility, and ease of use of the study medications [23].

### Dulaglutide versus glargine (combination therapy study)

Dulaglutide was superior to glargine in the PAM-D21-J Convenience/Flexibility, Perceived Effectiveness, and Emotional Effects subscales and in the IDMQ-J Satisfaction and Blood Glucose Control subscales. No significant differences were observed for dulaglutide compared to glargine in the IDMQ-J Ease of Use or Lifestyle Impact subscales as were observed in the monotherapy study for dulaglutide compared to liraglutide. This result suggests that the Ease of Use subscale may not be able to accurately measure patients’ perceptions about differences in injection frequency, most likely because the Ease of Use subscale includes an abstract question about convenience but no specific items about injection frequency. The statistically significant differences in the PAM-D21-J Perceived Effectiveness subscale and the IDMQ-J Blood Glucose Control subscale may be explained by the better glycemic control in the dulaglutide group compared to the glargine group. The better clinical outcomes with dulaglutide compared to glargine may also have affected the psychological perception of treatment with dulaglutide, resulting in a higher score in the PAM-D21-J Emotional Effects subscale for dulaglutide compared to glargine, which is consistent with a previous report about the relationship between glycemic control and quality of life [23].

### Dulaglutide versus both comparators

Although once-weekly dulaglutide was statistically superior to both once-daily comparators in the IDMQ-J Treatment Satisfaction subscale, it is unknown how much injection frequency affected patients’ assessments of treatment satisfaction for each medication. In addition, the clinical relevance and interpretation of the treatment differences in the scores between dulaglutide and the comparators is unclear, and further research is required to interpret the differences in detail in real-world settings, for instance by measuring treatment adherence anchored to treatment satisfaction.

We expected to observe a greater influence of injection frequency on patient perceptions of convenience/ease of use of the treatments. Although dulaglutide was statistically superior to both active comparators (liraglutide and glargine) in the PAM-D21-J Convenience/Flexibility subscale, the differences in the scores were smaller than expected based on the different injection frequencies (once weekly vs. once daily). As was discussed previously, it appears that the wording of items in the PAM-D21-J Convenience/Flexibility and IDMQ-J Ease of Use subscales substantially affected patient responses, and it seems that these questionnaires may not be specific enough to capture the additional burdens placed on patients with once-daily medication dosing compared to once-weekly dosing.

Mean EQ-5D-5L scores at baseline in this clinical trial setting were numerically slightly higher, but consistent with those observed in previous research in Japan [24].

Results of PRO questionnaires completed by patients treated with dulaglutide 0.75 mg in the global dulaglutide AWARD studies were generally positive for dulaglutide; in particular, it improved treatment satisfaction based on the Diabetes Treatment Satisfaction Questionnaire status version (DTSQs) [16]. In the AWARD-1 study, once weekly dulaglutide 0.75 mg and 1.5 mg both resulted in significantly greater improvement in all DTSQs subscale scores compared to exenatide twice daily at both 26 and 52 weeks [25]. In the AWARD-3 study, no statistically significant differences were observed between the dulaglutide 0.75 mg and 1.5 mg groups and metformin in total treatment satisfaction based on the DTSQs at either 26 or 52 weeks.

### Limitations

These instruments were not psychometrically validated and were studied in clinical trial settings; thus, the results obtained in a real-world setting may be different. In addition, the relatively brief duration of the studies in comparison to the long-term treatment required for diabetes should be considered when evaluating these results.

Because these studies were designed to compare dulaglutide to active comparators (liraglutide or glargine) in an open-label fashion (dulaglutide and placebo were blinded in the monotherapy study), the results of the PRO instruments may have been biased. In addition, market formulations were used for the active comparators, whereas a prefilled syringe designed for use in clinical trials was used for dulaglutide and placebo; therefore, because dulaglutide is marketed as a single-dose pen, the interpretation of some of the PRO results, such as findings related to convenience of the treatments, may be challenging. Future research is necessary to evaluate patient perception of the marketed single-dose dulaglutide pen compared to comparators’ devices.

Some of the questions in the questionnaires may not have been applicable to these study medications. For example, “Easy to carry for use away from home” from the IDMQ-J (Lifestyle Impact subscale) is relevant for injectable medications that are administered frequently but may not be relevant for once-weekly medications such as dulaglutide.

Finally, it appeared that the PAM-D21-J and IDMQ-J may not be able to appropriately evaluate patients’ feelings about frequency of administration of antidiabetic medications: for example, dulaglutide significantly improved the PAM-D21-J Convenience/Flexibility subscale and the IDMQ-J Ease of Use subscale compared to placebo even though both medications were administered at the same frequency with the same device. It is believed that perhaps the PRO results were confounded by greater improvements in glycemic control in the dulaglutide group. Future research to develop PRO instruments that will more efficiently elicit and assess patients’ feelings about dosing frequency (eg, once weekly vs. once daily) would be very useful.

## Conclusions

Overall, after administration of once-weekly dulaglutide 0.75 mg to Japanese patients with T2D for 26 weeks, patient-reported health outcomes were generally positive versus the three comparator treatments (liraglutide, glargine, and placebo), suggesting increased treatment satisfaction through better blood glucose control and convenience/flexibility and reduced negative emotional effects of diabetes.

## Additional files

Additional file 1: The Perceptions About Medications-Diabetes 21 Questionnaire - Japanese version (PAM-D21-J). (PDF 61 kb)

Additional file 2: Injectable Diabetes Medication Questionnaire - Japanese version (IDMQ-J). (PDF 22 kb)

Additional file 3: Ethics review boards (ERBs) which approved the monotherapy study. (PDF 15 kb)

Additional file 4: Ethics review boards (ERBs) which approved the combination study. (PDF 14 kb)

## Abbreviations

ANOVA: Analysis of variance
BMI: Body mass index
CI: Confidence interval
DTSQs: Diabetes Treatment Satisfaction Questionnaire status version (DTSQs)
FAS: Full analysis set
GLP-1: Glucagon-like peptide-1
HbA1c: Glycated hemoglobin
IDMQ-J: Injectable Diabetes Medication Questionnaire - Japanese version
IDSQ-J: Insulin Delivery System Questionnaire - Japanese version
LOCF: Last observation carried forward
LSM: Least-square means
OHA: Oral hypoglycemia agent
PAM-D: Perception About Medications-Diabetes Questionnaire
PAM-D21-J: The Perceptions About Medications-Diabetes 21 Questionnaire, Japanese version
PRO: Patient-reported outcome
SE: Standard error
VAS: Visual analog scale
