# Supplementary material for: Evaluation of the impact of once weekly dulaglutide on patient-reported outcomes in Japanese patients with type 2 diabetes: comparisons with liraglutide, insulin glargine, and placebo in two randomized studies
Source: Health Qual Life Outcomes. 2017 Jun 12;15:123. doi: 10.1186/s12955-017-0696-7 (PMC5468988; doi:10.1186/s12955-017-0696-7)
Supplement: Supplementary file 1 — The Perceptions About Medications-Diabetes 21 Questionnaire - Japanese version (PAM-D21-J). (PDF 61 kb) [file 12955_2017_696_MOESM1_ESM.pdf]

Perceptions About Medications-Diabetes 21 Japanese version (PAM-D21-J) Questionnaire

Below are some general statements that people with diabetes might mention about their diabetes medications. In the past 4 weeks, how often have the following statements about taking your injectable diabetes medication applied to you? Please circle only one number on each line.

	**All of the time**	**Most of the time**	**Some of the time**	**None of the time**
The frequency with which I have to take my injectable diabetes medication is acceptable to me.	1	2	3	4
The amount of my new injectable medication that I need to take is acceptable to me.	1	2	3	4
The timing of taking my injectable diabetes medication is acceptable to me.	1	2	3	4
I feel like my injectable diabetes medication is….	**All of the time**	**Most of the time**	**Some of the time**	**None of the time**
working well for me.	1	2	3	4
making me feel better.	1	2	3	4
keeping my blood sugars stable.	1	2	3	4

Persons with diabetes often talk about emotional side effects from taking diabetes medications. How often, in the past 4 weeks, have you felt the following emotions as a result of taking your injectable diabetes medication? Please circle only one number on each line.

	**All of the time**	**Most of the time**	**Some of the time**	**None of the time**
Forgetful	1	2	3	4
Irritable	1	2	3	4
Frustrated	1	2	3	4
Nervous	1	2	3	4
Depressed	1	2	3	4

Copyright © 2016 Eli Lilly and Company
The PAM-D21-J is copyrighted by Eli Lilly and Company.
For permission to reproduce or use the PAM-D21-J, please contact copyright@lilly.com.

Persons with diabetes often talk about physical side effects from taking diabetes medication. In the past 4 weeks, how often have you felt the following physical side effects and problems as a result of taking your injectable diabetes medication? If you experienced the side effect at all in the past 4 weeks how much were you bothered by the side effect? Please circle only one number on each line.

	**In the past 4 weeks,** have you felt the following physical side effects or problems **as a result of taking your injectable diabetes medication?**		**If you experienced this side effect in the past 4 weeks, how much were you bothered by the side effect?** *If you answered "No" that you have not experienced this side effect, then please leave this question blank.*				
	**No**	**Yes**	**Not at all**	**A little bit**	**Moderately**	**Quite a bit**	**Extremely**
Feet or hands swelling	0	→	1	2	3	4	5
Headaches	0	→	1	2	3	4	5
Weight gain	0	→	1	2	3	4	5
Dizziness or lightheadedness	0	→	1	2	3	4	5
Bloating or gas	0	→	1	2	3	4	5
Diarrhea	0	→	1	2	3	4	5
Excessive sweating	0	→	1	2	3	4	5
Itching	0	→	1	2	3	4	5
Upset stomach (nausea)	0	→	1	2	3	4	5
Sleepy	0	→	1	2	3	4	5

Copyright © 2016 Eli Lilly and Company
The PAM-D21-J is copyrighted by Eli Lilly and Company.
For permission to reproduce or use the PAM-D21-J, please contact copyright@lilly.com.
