# Supplementary material for: Evaluation of the impact of once weekly dulaglutide on patient-reported outcomes in Japanese patients with type 2 diabetes: comparisons with liraglutide, insulin glargine, and placebo in two randomized studies
Source: Health Qual Life Outcomes. 2017 Jun 12;15:123. doi: 10.1186/s12955-017-0696-7 (PMC5468988; doi:10.1186/s12955-017-0696-7)
Supplement: Supplementary file 2 — Injectable Diabetes Medication Questionnaire - Japanese version (IDMQ-J). (PDF 22 kb) [file 12955_2017_696_MOESM2_ESM.pdf]

Injectable Diabetes Medication Questionnaire Japanese version (IDMQ-J)

1. Based on your experience during the past 4 weeks, how satisfied were you with your injectable diabetes medication that you used in this study?

Please circle the number that best indicates your level of satisfaction.

Very dissatisfied	Dissatisfied	Somewhat dissatisfied	Neither satisfied nor dissatisfied	Somewhat satisfied	Satisfied	Very satisfied
1	2	3	4	5	6	7

2. Based on your experience during the past 4 weeks, to what extent do you agree that your injectable diabetes medication that you used in this study has these features?

For each feature, please circle the number that best indicates your level of agreement.

Features	Strongly disagree	Disagree	Slightly disagree	Neither agree nor disagree	Slightly agree	Agree	Strongly agree
a) Easy to get the dose needed.	1	2	3	4	5	6	7
b) Easy to control my blood sugar.	1	2	3	4	5	6	7
c) Easy to carry for use away from home.	1	2	3	4	5	6	7
d) Easy to incorporate into my daily life.	1	2	3	4	5	6	7
e) Convenient for me to use.	1	2	3	4	5	6	7
f) Not stressful for me to use.	1	2	3	4	5	6	7

Copyright © 2016 Eli Lilly and Company
The IDMQ-J is copyrighted by Eli Lilly and Company.
For permission to reproduce or use the IDMQ-J, please contact copyright@lilly.com.

3. Based on your experience during the past 4 weeks, to what extent do you believe that the injectable diabetes medication that you used in this study helped control your blood sugars?

For each question, please circle the number that best indicates how much of the time you believe your blood sugar was controlled.

How much of the time during the past 4 weeks:	All of the time	Most of the time	A good bit of the time	Some of the time	A little of the time	None of the time
a) Did you feel that your diabetes was well controlled?	1	2	3	4	5	6
b) Did you feel that your blood sugars were too high?	1	2	3	4	5	6
c) Did you feel that your blood sugars were too low?	1	2	3	4	5	6

4. Based on your experience during the past 4 weeks, to how willing would you be to continue to use the injectable diabetes medication that you used in this study.

Please circle the number that best indicates your level of willingness.

Definitely unwilling	Mostly unwilling	Don't know	Mostly willing	Definitely willing
1	2	3	4	5

Copyright © 2016 Eli Lilly and Company
The IDMQ-J is copyrighted by Eli Lilly and Company.
For permission to reproduce or use the IDMQ-J, please contact copyright@lilly.com.
