# Supplementary material for: Evaluation of the impact of once weekly dulaglutide on patient-reported outcomes in Japanese patients with type 2 diabetes: comparisons with liraglutide, insulin glargine, and placebo in two randomized studies
Source: Health Qual Life Outcomes. 2017 Jun 12;15:123. doi: 10.1186/s12955-017-0696-7 (PMC5468988; doi:10.1186/s12955-017-0696-7)
Supplement: Supplementary file 3 — Ethics review boards (ERBs) which approved the monotherapy study. (PDF 15 kb) [file 12955_2017_696_MOESM3_ESM.pdf]

Ethics review boards (ERBs) which approved the monotherapy study

Aino clinic IRB
AMC Nishi Umeda Clinic IRB
H.E.C Science Clinic IRB
Hyogo College of Medicine Hospital Institutional Review Board
IRB of Japanese Red Cross Fukuoka Hospital
Jinnouchi Hospital IRB
Kanazawa Medical Clinic Institutional Review Board
Kanazawa University Hospital Institutional Review board
Kanto Rosai Hospital IRB
Kimura Hospital Medical Corporation Yuwakai Institutional Review Board
Manda Memorial Hospital Institutional Review Board
Matsumoto Nakagawa Hospital Institutional Review Board
Medical Corporation Heishinkai OPHAC Hospital IRB
Medical Corporation Shintokai Yokohama Minoru Clinic Institutional Review Board
Okada Clinic IRB
OKUGUCHI Clinic of Internal Medicine Institutional Review Board
P-One Clinic Institutional Review Board
Sapporo City General Hospital Institutional Review Board
Shinagawa East one Medical Clinic Institutional Review Board
Takatsuki Red Cross Hospital IRB
The Institute for Adult Diseases, Asahi Life Foundation IRB
The Japanese Association for the Promotion of State-of-the-Art in Medicine 2nd Institutional Review Boards
Tokyo Medical University Hospital IRB
Tokyo-Eki Center-building Clinic Institutional Review Board
Umezu Clinic, Medical Corporation Houmankai IRB
