# Supplementary material for: Evaluation of the impact of once weekly dulaglutide on patient-reported outcomes in Japanese patients with type 2 diabetes: comparisons with liraglutide, insulin glargine, and placebo in two randomized studies
Source: Health Qual Life Outcomes. 2017 Jun 12;15:123. doi: 10.1186/s12955-017-0696-7 (PMC5468988; doi:10.1186/s12955-017-0696-7)
Supplement: Supplementary file 4 — Ethics review boards (ERBs) which approved the combination study. (PDF 14 kb) [file 12955_2017_696_MOESM4_ESM.pdf]

Ethics review boards (ERBs) which approved the combination study

Abe Clinic IRB
Aichi Medical Association Institutional Review Board
AMC Nishi Umeda Clinic IRB
Asama General Hospital Institutional Review Board
Azumi General Hospital Institutional Review Board
Ehime Prefectural Central Hospital Institutional Review Board
Hakujyujikai Sasebo Chuo Hospital IRB
Hatamoto Institutional Review Board
Iryo Houjin Hakuunkai Ryoya Komatsu Clinic Institutional Review Board
JR Tokyo General Hospital Institutional Review Board
Kanazawa Medical Clinic Institutional Review Board
Kimura Hospital Medical Corporation Yuwakai Institutional Review Board
Kitano Hospital, the Tazuke Kofukai Medical Research Institute IRB
Komoro Kosei General Hospital Institutional Review Board
Kumamoto University Hospital Institutional Review Board
Kyoto University Hospital Institutional Review Board
Manda Memorial Hospital Institutional Review Board
Matsumoto Nakagawa Hospital Institutional Review Board
Medical Corporation Kojinkai Sapporo Skin Clinic IRB
Oki Medical Clinic Institutional Review Board
Shinagawa East one Medical Clinic Institutional Review Board
Takatsuki Red Cross Hospital IRB
Takeda Hospital Group Institutional Review Board
Tokushukai Group Institutional Review Board
Tokyo-Eki Center-building Clinic Institutional Review Board
Umezu Clinic, Medical Corporation Houmankai IRB
Yamaguchi University Hospital Institutional Review Board
